# Identifying patients with diabetes and the earliest date of diagnosis in real time: an electronic health record case-finding algorithm

**DOI:** 10.1186/1472-6947-13-81

**Published:** 2013-08-01

**Authors:** Anil N Makam, Oanh K Nguyen, Billy Moore, Ying Ma, Ruben Amarasingham

**Affiliations:** 1 Division of General Internal Medicine, University of California San Francisco, Box 1211, Laurel Heights Campus, Room 383, 3333 California St., San Francisco, CA 94143, USA; 2 Parkland Center for Clinical Innovation, Dallas, TX, USA; 3 Division of General Internal Medicine, University of Texas Southwestern Medical Center, 5323 Harry Hines Blvd., Dallas, TX 75390, USA

## Abstract

**Background:**

Effective population management of patients with diabetes requires timely recognition. Current case-finding algorithms can accurately detect patients with diabetes, but lack real-time identification. We sought to develop and validate an automated, real-time diabetes case-finding algorithm to identify patients with diabetes at the earliest possible date.

**Methods:**

The source population included 160,872 unique patients from a large public hospital system between January 2009 and April 2011. A diabetes case-finding algorithm was iteratively derived using chart review and subsequently validated (n = 343) in a stratified random sample of patients, using data extracted from the electronic health records (EHR). A point-based algorithm using encounter diagnoses, clinical history, pharmacy data, and laboratory results was used to identify diabetes cases. The date when accumulated points reached a specified threshold equated to the diagnosis date. Physician chart review served as the gold standard.

**Results:**

The electronic model had a sensitivity of 97%, specificity of 90%, positive predictive value of 90%, and negative predictive value of 96% for the identification of patients with diabetes. The kappa score for agreement between the model and physician for the diagnosis date allowing for a 3-month delay was 0.97, where 78.4% of cases had exact agreement on the precise date.

**Conclusions:**

A diabetes case-finding algorithm using data exclusively extracted from a comprehensive EHR can accurately identify patients with diabetes at the earliest possible date within a healthcare system. The real-time capability may enable proactive disease management.

## Background

Practice redesign efforts are shifting the paradigm from volume to value in healthcare in part by emphasizing care coordination, population health, and performance reporting. To this end, the National Committee for Quality Assurance (NCQA) requires practices to use patient tracking, disease registries and certified electronic health records (EHR) in order to qualify for patient-centered medical home (PCMH) and accountable care organization (ACO) accreditation [1,2].

Diabetes is well-suited to the principles of the PCMH and ACO, given that it affects 25.8 million people, [3] costs $174 billion annually, [4] and despite well-established and effective guidelines only 45% of diabetics receive recommended care [5,6]. Effective practice redesign efforts aimed at improving the care of diabetes will require proactive identification of patients with diabetes to narrow the knowledge-to-action gap. While existing diabetes case-finding algorithms are able to accurately identify patients with diabetes, [7-17] such algorithms rely on historical rather than real-time data. As a result, there may be a lag time between when a patient receives a diagnosis of diabetes in the clinical setting compared to when the patient is identified as a diabetic by a case-finding algorithm for the purpose of population management. Because preventing complications of diabetes depends critically on timely intervention, [18] this lag impedes the potential for case-finding algorithms to significantly affect prevention of such complications across diabetic populations.

No published case-finding algorithm, that we know of to date, takes advantage of a comprehensive EHR that would allow for a fully automated and electronic system obviating the need for manual data entry, and importantly, real-time availability for data capture and identification [19]. Therefore, this study aims to derive and validate an electronic case-finding model (e-model) that could be used in real-time to identify patients who meet criteria for diabetes at the earliest possible date based on EHR data within a healthcare system.

## Methods

### Study population

The e-model was developed using historical data extracted from an EHR (Epic Systems Corporation, Verona, WI) deployed across inpatient and outpatient settings within Parkland Health & Hospital System (PHHS), a large urban safety-net health system in Dallas, TX. We used data from 160,872 unique adult patients (age ≥ 18 years) who had a first encounter within PHHS between January 1, 2009 and April 1, 2011.

### Definition of algorithm variables

To determine the criteria used by the e-model to identify diabetes, we used a combination of diagnostic criteria from the American Diabetes Association (ADA) [5] and data elements identified by a panel of physician health service researchers, including a board certified endocrinologist (AM, CR, RA). Only those data elements which were routinely documented and extractable from structured data fields (e.g., encounter diagnosis, past medical history, problem list, medications, and laboratory results) within the EHR were included as variables in the e-model.

### Derivation of E-model

We used a point-based algorithm to identify the presence of diabetes and to determine the date that a diagnosis could have been made through information in the EHR. Each variable in the e-model was initially assigned a fractional point value, proportionate to its perceived relative contribution in diagnosing diabetes. Point totals of 0, between 0 and 1, and ≥ 1 were set as thresholds for e-model determination of ‘no diabetes,’ ‘possible diabetes,’ and ‘diabetes,’ respectively. The e-model determined the diagnosis date as the date when accumulated points reached or exceeded a threshold value of 1.

The perceived relative contribution of each variable was determined based on ADA diagnostic criteria, [5] existing diabetes case-finding algorithms, [10] and expert opinion. For example, since the ADA requires two fasting blood glucose values of ≥ 126 mg/dL for the diagnosis of diabetes, the presence of a single fasting blood glucose value of ≥ 126 mg/dL was assigned a point value of 0.5, such that two fasting blood glucose values would give an individual a total of 1 point for an e-model identification of ‘diabetes.’

**Figure 1:**
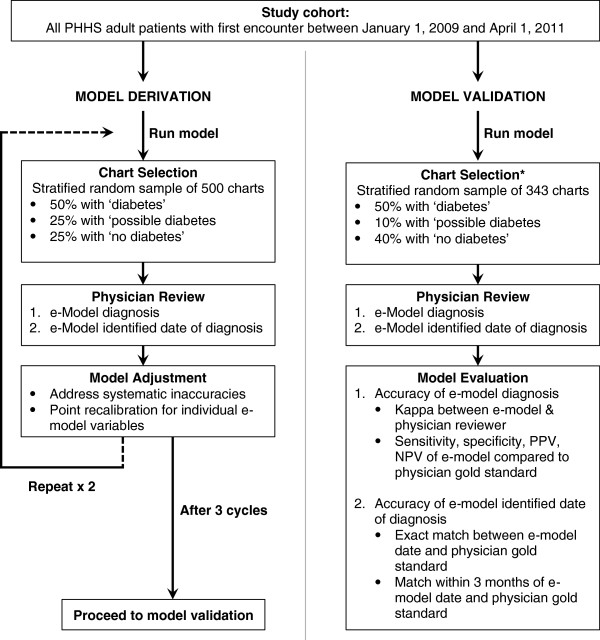
Electronic diabetes case-finding model derivation and validation flowchart. * Charts used in derivation were excluded from the validation cohort.

Point assignments were subsequently recalibrated through a clinically-guided strategy, consisting of an iterative, three-stage evaluation process (Figure 1). At each stage, a stratified random sample of up to 500 charts, with 50% ‘diabetes,’ 25% ‘possible diabetes,’ and 25% ‘no diabetes’ as determined by the e-model, underwent unblinded chart review by a physician to evaluate the accuracy of the e-model identification of diabetes and diagnosis date. To allow better evaluation of e-model performance, the ‘no diabetes’ group was restricted to individuals 50 years or older since the incidence of diabetes is strongly associated with age and may increase the potential to identify false negatives. To allow for evaluation of the accuracy of the e-model diagnosis date, 50% of the ‘diabetes’ group was selected to have accumulated ≥ 1 point(s) on a date more recent than the date of the first encounter. This allowed for a potentially earlier date of diagnosis to be determined by chart review.

Points for individual variables were reweighted after each stage based on commonly recurring e-model inaccuracies and were finalized after three successive stages (Table 1).

**Table 1:** Variables included in the electronic diabetes case-finding model

**Identification variable**	Criteria^*^	**Encounter location**	**Point value**
ICD-9 encounter code	250.xx	Inpatient or outpatient	0.75
Hemoglobin A1c	≥ 6.5%	Inpatient or outpatient	1.00
Fasting Blood Glucose	≥ 126 mg/dL	Outpatient only	0.50
Random blood glucose	≥ 200 mg/dL	Outpatient only	0.50
2-hour OGTT	≥ 200 mg/dL	Inpatient or outpatient	0.75
Problem list or PMH	250.xx	Inpatient or outpatient	0.40
Diabetes medication^**^	Present	Outpatient only	1.00
Metformin	Present	Outpatient only	0.75

Abbreviations: *ICD-9:* International Classification of Diseases 9, *OGTT:* oral glucose tolerance test, *PMH:* past medical history, *mg:* milligram, *dL:* deciliter.

^*^Criteria are counted only once except for ICD-9 codes (maximum twice) and random and fasting blood glucose (maximum twice each) as long as repeated glucose values are ≥ 3 months apart.

^**^Insulin, sulfonylurea, thiazolidinedione, alpha glucosidase, DPP-4 inhibitor, meglitinide, amylin mimetic, incretin mimetic, combination medication.

Through the derivation process, we adjusted the point values for diabetes medication, problem list and past medical history, and ICD-9 encounter diagnosis. The presence of any diabetes medication was initially assigned a point value of 1, since we considered this a surrogate for the presence of diabetes given that the primary and often only indication is the treatment of hyperglycemia. However, during chart review, metformin was found to be occasionally prescribed for pre-diabetes and polycystic ovarian syndrome, and individuals with only the presence of metformin were incorrectly identified by the e-model as ‘diabetes.’ Therefore, the point value for metformin was decreased to 0.75. The point value for the presence of diabetes in the past medical history or problem list fields were reduced to 0.4 because the data in these fields were found to be often inaccurate and outdated. Lastly, a single ICD-9 encounter diagnosis in the absence of other variables incorrectly identified patients as having diabetes in most cases. Existing case-finding algorithms have also found the presence of two ICD-9 codes across outpatient and inpatient settings to be highly sensitive and more specific than a single code for the diagnosis of diabetes [20]. Therefore, the encounter diagnosis variable was adjusted from 1 to 0.75 points.

### Validation of E-model

To validate the e-model, we compared the e-model identification of ‘diabetes,’ ‘possible diabetes’ and ‘no diabetes’ and date of diagnosis to the gold standard of physician chart review. Based on a conservative estimate of 70% sensitivity +/− 10% error at a two-sided alpha of 0.05 and power of 80%, we determined that a stratified random subset of 343 patients (50% ‘diabetes,’ 10% ‘possible’ diabetes’, and 40% ‘no diabetes’) was needed to adequately validate the e-model. Charts reviewed during the derivation process were not included in the validation cohort.

Blinded chart review for an initial 50 patients was completed by two board-certified internists working independently (AM and ON). Inter-rater agreement between reviewers for the classification of diabetes status was 0.80 with a linear weighted kappa statistic and 0.94 for the exact diagnosis date when both reviewers agreed that diabetes was present (n = 19). The remaining charts were reviewed by a single physician after establishing adequate inter-rater reliability.

To examine model performance, the “possible diabetes” group was combined with the “no diabetes” group to allow for a dichotomous classification. Two sensitivity analyses were performed on the validation cohort to 1) determine the optimal point threshold for identification of diabetes and 2) determine the effect of an alternate dichotomous grouping on e-model performance. For the first sensitivity analysis, we varied the point threshold for the identification of diabetes to test for the optimal cutoff point to maximize e-model performance. For the second, we combined the “possible diabetes” group with the “diabetes” group to examine the effect on e-model sensitivity and specificity.

We then evaluated the performance of the e-model on correct identification of the earliest diagnosis date. The e-model date was considered to be in agreement with the physician date of diagnosis if it was within a 3 month window of the physician-determined date. The time interval of 3 months was chosen based on the ADA consensus opinion which recommends repeat glycemic testing in 3 months for newly diagnosed or poorly controlled diabetes patients [5]. Lastly, we compared the performance of the e-model in identifying the date of diagnosis to the performance of a simplified claims-based case-finding algorithm that would not require the presence of an EHR. This method utilized the presence of only two diabetes encounter codes (ICD-9 250) to identify the diagnosis date.

### Statistical analysis

The diagnostic performance of the e-model on identification of diabetes status was evaluated first by the inter-rater agreement between e-model classification and physician classification of diabetes status using the kappa statistic and second, by using sensitivity, specificity, positive predictive value (PPV), and negative predictive value (NPV). The optimal point threshold was evaluated by the receiver-operator curve. We evaluated the performance of the e-model on correct identification of the diagnosis date by the inter-rater agreement between the e-model date of diagnosis and physician determination of date of diagnosis for individuals identified as ‘diabetes’ by the physician.

Analyses were conducted using STATA statistical software (version 12.0; STATA Corp, College Station, TX). The University of Texas Southwestern Medical Center Institutional Review Board approved the research protocol.

## Results

Using the e-model to characterize the overall study cohort, the source population included 14,025 (8.7%) patients identified as having ‘diabetes,’ 1,882 (1.2%) patients as ‘possible diabetes,’ and 144,965 (90.1%) patients as ‘no diabetes.’ In the overall diabetic population (n = 14,205), the mean age was 52 years (+/− 13 years), 44% were Hispanic, 27% black, 22% white, 53% male, 68% had a primary payer of self-pay or charity, and the mean number of healthcare encounters was 6.5. Of the 1,500 charts for model derivation, 83 were excluded because of duplicated patients or age less than 18 years. Of the 343 charts for validation, 2 were excluded from analysis because the chart did not exist (n = 1) or was a duplicate (n = 1). Patients in the derivation and validation cohorts were similar with respect to age, race and ethnicity, sex, and primary payer, but patients in the derivation group had a slightly greater number of encounters over a one-year period (Table 2).

**Table 2:** Baseline cohort characteristics for the electronic diabetes case-finding model*

**Characteristics**	**Derivation**	**Validation**	P-value**
n	1417	341	
Age, mean years (SD)	49.3 (14.4)	45.0 (14.6)	<.001
Race/ethnicity,%			.83
Hispanic	44	40	
White	23	24	
Black	25	27	
Other	9	9	
Male,%	47	52	.13
Primary payer,%			.15
Commercial	18	16	
Medicare	9	6	
Medicaid	13	13	
Self-pay	30	29	
Charity	30	36	
Encounters, mean no. (SD)			
All	7.86 (9.03)	6.65 (9.06)	.01
Primary care	2.25 (3.90)	1.99 (3.60)	.45
Specialty care	4.31 (6.86)	3.62 (7.08)	.02
Urgent care and ED	0.81 (2.58)	0.61 (0.89)	.48
Inpatient	0.49 (0.97)	0.43 (0.88)	.25

*Derivation and validation cohorts defined as per Figure 1.

**Student *t*-test for age; *χ*^2^ tests for race/ethnicity, male sex, and primary payer; Wilcoxon rank-sum test for encounters.

### E-model performance on identification of diabetes

The kappa statistic between the e-model and physician reviewer on the question of whether diabetes was present was 0.76 (p < 0.001) with 86% overall agreement. Combining the “possible diabetes” group with the “no diabetes” group, the sensitivity, specificity, positive predictive value, and negative predictive value of the e-model were 97%, 90%, 89%, and 97% respectively. Alternatively, when the “possible diabetes” group was combined with the “diabetes” group, the sensitivity and specificity of the e-model were 99% and 81% respectively.

**Figure 2:**
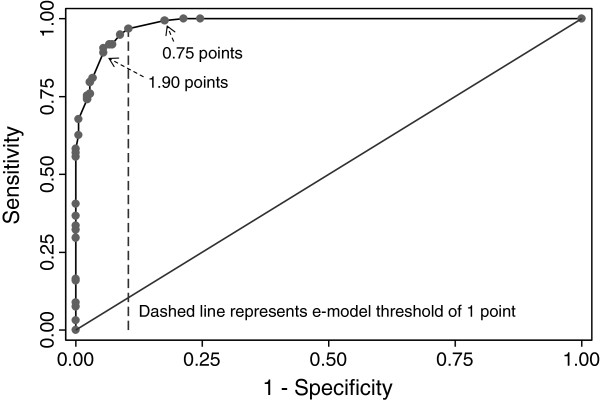
Receiver operating characteristic curve for the electronic diabetes case-finding model identification of diabetes compared to physician review by different point thresholds (C statistic 0.98).

The performance of the e-model by different point thresholds for the identification of diabetes corroborated using a threshold of 1 point to optimize sensitivity and specificity (Figure 2).

### E-model performance on earliest date of diagnosis

**Figure 3:**
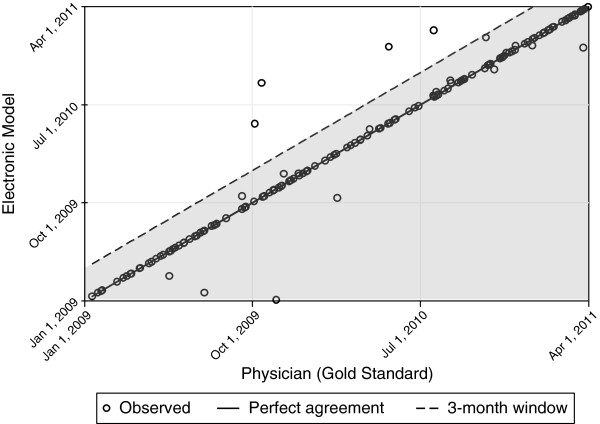
**Comparison of the date of diagnosis of diabetes within a healthcare system as ascertained by the electronic diabetes case-finding model and physician reviewer.** Observations below and to the right of the dashed line (shaded area) are within the allowed 3-month window for agreement.

The kappa score between e-model and physician on the date of diagnosis was 0.94 with agreement on the exact date in 76% of the cases. Among the cases where both the physician and e-model made a diagnosis of diabetes, only 4 observations (2.6%) were diagnosed by the e-model more than 3 months after the correct date (Figure 3).

However, a more simplified claims-based algorithm that used only two ICD-9 encounter diagnoses (e.g. the diagnosis date equals the date of the second ICD-9 code) did not accurately identify the diagnosis date compared to a physician reviewer. Using this approach, the kappa statistic was 0.62 with only 4.6% agreement on the exact date. Notably, 27% of the cases (n = 33) did not have a second ICD-9 encounter code during the study period.

## Discussion

We developed an electronic case-finding algorithm that accurately identified patients with diabetes at their earliest possible date within a healthcare system using data extracted from an EHR. The performance of our model in identifying patients with diabetes is comparable to other diabetes case-finding algorithms [10-17]. However, the distinct advantage of our automated, real-time algorithm is the timely recognition of diabetes. Relying on only two ICD-9 encounter codes to establish the diagnosis date, a quarter of the cases in our cohort would have been missed and another 11% would have had a delayed diagnosis. By using multiple data elements we were able to identify the date of diagnosis within three months of a physician’s chart review date in 94% of case, with three-quarters of cases having a perfect date match.

Achieving early glycemic control in patients with newly diagnosed diabetes reduces the risk of microvascular complications, myocardial infarction, and all-cause mortality [18]. Attaining the benefits of instituting early treatment requires timely diagnosis. In the ARIC cohort, a population-based prospective study of middle-aged adults, Samuels et al. found that even with an effective screening program the median delay from the onset of diabetes to physician diagnosis was 2.4 years, with more than 7% of incident cases remaining undiagnosed for at least 7.5 years [21]. In addition, delayed diagnoses are more widespread in safety-net settings where patients may have more fragmented, episodic care [22]. Real-time, automated patient identification and tracking can help healthcare systems close this gap and facilitate the delivery of timely, effective therapy at the point-of-care at the earliest possible date [19].

Improving care for diabetics is increasingly important for healthcare systems in today’s pay-for-performance climate. The high cost, rising prevalence, and documented quality gap has positioned diabetes in the forefront of policies benchmarking performance. To qualify for financial incentives and avoid public scrutiny, healthcare systems are increasingly faced with the challenge to achieve acceptable rates in their diabetic population for targeted metrics such as glycated hemoglobin, low-density lipoprotein, and screening for microalbuminuria. Our electronic case-finding algorithm, which leverages real-time data in the EHR, can enable proactive management of these quality measures. Healthcare systems may additionally apply this algorithm to provide feedback to providers on the quality of their care, generate letters to patients, identify underperforming clinics for quality improvement initiatives, link clinical decision support tools to inform decision making at the point-of-care, and risk stratify diabetic patients to direct limited resources to patients at greatest risk for developing complications.

Our study has several limitations. First, as with other registries, the limitations of miscoding and misclassification prohibited subtype distinction between type 1, type 2, and secondary diabetes [23]. Second, due to limits in study costs, we established an enriched prevalence of diabetes of 50% in our validation cohort to reduce the number of charts required for manual chart review. While this inflated the positive and negative predictive values, the sensitivity and specificity of our algorithm remained unaffected and were comparable to other diabetes case-finding algorithms. Third, by using the e-model algorithm to select our validation cohort, we were unable to evaluate how individual data elements performed in identifying diabetes. Fourth, the direct applicability of our algorithm to other settings is unknown because of differences in practice style, EHR integration across outpatient and inpatient settings, and EHR documentation. Systems with greater accuracy in EHR documentation may need to increase the relative weight of the problem list and past medical history field to maximize the model’s performance. With proper weight adjustments we expect our algorithm to be suitable to a wide range of healthcare settings. Automated machine learning techniques may provide approaches to model adjustment that could minimize manual recalibration and allow larger scales of dissemination. Lastly, in clinical settings transitioning from paper-based records to an EHR, the e-model may not accurately distinguish between newly established versus preexisting cases of diabetes within a healthcare system [24].

## Conclusion

Our electronic case-finding algorithm can accurately identify patients with diabetes at the earliest possible date within a healthcare system. We believe this algorithm can be used by healthcare systems with comprehensive EHRs to build real-time diabetes identification systems. This is foundational to diabetes “system awareness,” or building information systems that are able to construct and maintain awareness of a patient’s status across time, setting, provider, and context.

## Competing interests

The authors’ declare that they have no competing interests.

## Authors’ contributions

All authors in this study have been involved in concept and design, critical revision of the manuscript for important intellectual content, and giving final approval of the manuscript for submission. AM and RA designed the study. AM and ON served as physician chart reviewers. AM performed data analysis, interpretation and drafting of the manuscript, with significant contributions from ON, YM, and RM. YM participated in refinement of derivation process and data analytics. All authors’ read and approved the final manuscript.

## Pre-publication history

The pre-publication history for this paper can be accessed here:

http://www.biomedcentral.com/1472-6947/13/81/prepub
